# *In vitro* evidence of antioxidant and anti-inflammatory effects of a new nutraceutical formulation explains benefits in a clinical setting of COPD patients

**DOI:** 10.3389/fphar.2024.1439835

**Published:** 2024-08-20

**Authors:** Valentina Lazzara, Paola Pinto, Serena Di Vincenzo, Maria Ferraro, Filippo Catalano, Pietro Provinzano, Elisabetta Pace, Maria Rosaria Bonsignore

**Affiliations:** ^1^ Dipartimento Promozione della Salute Materno-Infantile di Medicina Interna e Specialistica di Eccellenza “G. D’Alessandro” (PROMISE), Università degli Studi di Palermo, Palermo, Italy; ^2^ Institute of Translational Pharmacology (IFT), National Research Council of Italy (CNR), Palermo, Italy; ^3^ PhD National Program in One Health Approaches to Infectious Diseases and Life Science Research, Department of Public Health, Experimental and Forensic Medicine, University of Pavia, Pavia, Italy; ^4^ Azienda Ospedaliera Ospedali Riuniti Villa Sofia Cervello, Palermo, Italy

**Keywords:** airway epithelial cells, cigarette smoke, inflammation, oxidative stress, antioxidant, nutraceutical formulation

## Abstract

**Background and Aim:** Increased oxidative stress within the airways is associated to epithelial damage and amplification of inflammatory responses that in turn contribute to Chronic Obstructive Pulmonary Disease (COPD) progression. This study was aimed to identify whether a new formulation of N-acetylcisteine (NAC), carnitine, curcumin and B2 vitamin could counteract oxidative stress and downstream pro-inflammatory events promoted by cigarette smoke extract (CSE) exposure in primary bronchial epithelial cells (PBEC), both submerged/undifferentiated (S-PBEC) and cultured at the air-liquid interface (ALI-PBEC).

**Methods:** PBEC were exposed to CSE with/without the new formulation or NAC alone and ROS production, IL-8 and IL-6 gene expression and protein release were evaluated.

**Results:** CSE increased ROS, IL-8 and IL-6 gene expression and protein release and the new formulation counteracted these effects. NAC alone was not effective on IL-8 and IL-6 release. The effects of a similar nutraceutical formulation were evaluated in COPD patients treated for six months. The results showed that the treatment reduced the concentration of IL-8 in nasal wash and improved quality of life.

**Conclusion:** The tested formulation, exerting antioxidant and anti-inflammatory effects, can preserve airway epithelial homeostasis and improve clinical symptoms in COPD.

## 1 Introduction

COPD is a chronic inflammatory pulmonary disease, which affects the lungs and bronchi, has a multifactorial etiology and a variable entity depending on its severity (Mannion et al., 2022). It is a long-lasting chronic condition whose damage is often irreversible, is characterized by symptoms such as dyspnea, cough, wheezing, excessive mucus production, weakness, and has two main phenotypes, emphysema and chronic bronchitis (Labaki and Rosenberg, 2020). Exacerbations in COPD may be frequent, and are also major phenotypic traits (Hurst et al., 2010; Agustí et al., 2023). The best known and most common cause of COPD is cigarette smoke, which irritates the mucous membranes favoring the onset of inflammatory processes (Aghapour et al., 2018). Cigarette smoke is known to be a major agent inducing oxidative stress and chronic lung inflammation (Sun et al., 2019), also leading to accelerated lung aging (Fairley et al., 2023). Oxidative stress is a key factor in the pathogenesis of COPD, especially in the exacerbation phases (Barnes, 2020), also increases chronic inflammation, stimulates fibrosis and emphysema, and in some cases even causes corticosteroid resistance (Bodas et al., 2017). In recent years, the field of nutraceuticals has acquired increasing importance. In fact, it is now widely demonstrated that diet and the use of nutritional supplements have a beneficial impact on human health, in the context of various pathologies, including respiratory diseases (Scoditti et al., 2019; Rahman et al., 2022). N-Acetylcysteine (NAC) is widely employed as mucolytic and antioxidant agents in several human diseases, in particular respiratory diseases, such as COPD (Pedre et al., 2021; Tenório et al., 2021). In patients with COPD, reduced levels of GSH have been demonstrated, while NAC contributes to restore and maintain the concentration of GSH high, ensuring its antioxidant actions (Rushworth and Megson, 2014; Aldini et al., 2018; Raghu et al., 2021). Several *in vitro* studies demonstrated a protective role of NAC against the mitochondrial oxidative damage, reducing the amount of reactive oxygen species (ROS) and restoring the normal function of the mitochondrial respiratory chain (Xiao et al., 2016; Liu et al., 2021; Li et al., 2022). Another important role of NAC is related to its anti-inflammatory activity, widely shown both *in vitro* and *in vivo* studies (Mohanty et al., 2021). Patients with COPD show a typical inflammatory asset in which levels of interleukin-6 (IL-6) and interleukin-8 (IL-8/CXCL8) are high, especially during exacerbations. IL-8 is released by bronchial epithelial cells and is a crucial cytokine for the activation of neutrophils in lungs (Pace et al., 2011). NAC is able to reduce gene expression, and thus protein release, of inflammatory cytokines such as TNF-α, IL-6, IL-1β and chemokines such as IL-8, through the downregulation of the nuclear transcription factor kappa B (NF-κB) (Radomska-Leśniewska et al., 2010; Zhang et al., 2019; Chi et al., 2022; Le et al., 2022). Numerous natural substances, in addition to traditional pharmacological therapies, have demonstrated interesting antioxidant, anti-inflammatory and anticancer effects. One of the most studied categories is that of phytocompounds (Mucha et al., 2021; Ożarowski and Karpiński, 2021), among them *Curcuma longa* exerts a relevant role. The derivative compound, curcumin, showed interesting antioxidant and anti-inflammatory effects in the context of several diseases, counteracting the oxidative damage and by reducing the inflammatory mediators (Lin et al., 2019; Mrityunjaya et al., 2020; Ran et al., 2021). Several *in vitro* studies have also demonstrated an antioxidant role of L-carnitine (Li et al., 2023). L-Carnitine (beta-hydroxy-gamma N-trimethyl amino-butyric acid) is a quaternary amine that mediates as an essential transport cofactor that transports fatty acids into the mitochondrial matrix and plays a critical role in fatty acid energy metabolism (Hoang et al., 2023). L-Carnitine is a water-soluble vitamin-like substance that occurs naturally in the human body (Talenezhad et al., 2020; Carrillo-González et al., 2023). In COPD, cachexia and muscle weakness are common systemic clinical manifestations besides pulmonary symptoms. According to some studies, L-carnitine deficiency occurs in COPD patients, and use of L-carnitine as a potential therapeutic agent for the long-term management of COPD patients has been proposed (Hoang et al., 2023). Indeed, L-carnitine reduces oxidative stress and inflammation by modulating efficient fatty acid oxidation (Pekala et al., 2011), functions that may reduce cytokine production in the inflammatory process and decrease myocyte damage in COPD patients (Krajcovicová-Kudlácková et al., 2000). Vitamins also play a crucial role in the proper functioning of the organism. Disorders of their levels, both deficiency or excess, promote the development of various diseases, including those of the respiratory system (Cheng et al., 2023). Reduced level of soft B vitamins have been documented in COPD patients, and supplementation of B vitamins could have a beneficial effect on oxidative stress in COPD patients (Kırkıl and Muz, 2008). Interestingly, studies have shown a promising improvement in alleviating systemic oxidative stress and clinical symptoms, and an improvement in health-related quality of life in subjects with chronic lung complications after supplementation with certain curcuminoids and piperine (Panahi et al., 2016). Piperine also has excellent therapeutic capabilities. Studies show that piperine in mice with COPD suppresses airway obstruction, oxidative stress and mitigates the increase in inflammatory cells and inflammatory biochemicals mediated by cigarette smoke (Arora et al., 2022). In light of this, the aim of our work was to evaluate the antioxidant and anti-inflammatory efficacy on human primary bronchial epithelial cells of a new nutraceutical formulation that associates NAC, already widely recognized and employed for its beneficial effects, with other natural substances: curcumin, carnitine, vitamin B2. The degree of mitochondrial oxidative stress and the gene expression and release of inflammatory cytokines such as IL-6 and IL-8 was evaluated on submerged/undifferentiated primary bronchial epithelial cells (S-PBEC) and then the results were confirmed in a more complex model of differentiated cells (ALI-PBEC) that more closely mirrors the *in vivo* conditions. Finally, clinical benefits as well as anti-inflammatory effects were also evaluated in COPD patients before and after 6 months treatment with a nutraceutical drug with curcumin, carnitine, acetyl-N-cysteine-L, vitamin B2, piperine.

## 2 Materials and methods

### 2.1 Human primary bronchial epithelial cells (PBECs) culture

Human primary bronchial epithelial cells (PBECs, ATCC-PCS-300-010) were purchased from American Type Culture Collection (ATCC, Rockville, MD, United States). PBECs were cultured submerged/undifferentiated (S-PBECs) to first evaluate markers and molecular mechanisms in a less complex *in vitro* model. PBECs were seeded on 6-well or 12-well plates previously coated with a collagen/fibronectin solution and grown in Bronchial Epithelial Cell Medium-basal (BEGM, LONZA Basel, Switzerland) and Dulbecco’s modified Eagle’s medium (DMEM) (Stemcell Technologies, Netherlands) mixture (1:1), supplemented with 12.5 mM Hepes, 1 mM Glutamax (Gibco, Thermo fisher scientific, Waltham, MA, United States), bronchial epithelial cell growth supplement (BEpiCGS; Sciencell, Sanbio, Netherlands), 100 U/mL penicillin and 100 μg/mL streptomycin (all from ScienCell, Sanbio, Netherlands) until confluence. To further investigate the same aspects in a more complex cellular model, PBECs were also cultured at the air–liquid interface (ALI-PBEC). In brief, 40,000 cells at passage two were seeded on 0.4 μm pore sized 12-well transwell membranes (Corning Costar, Glendale, AZ, United States), coated with a collagen/fibronectin solution. The cells were grown in the complete BEGM/DMEM medium (B/D), supplemented with 1 nM of the retinoic receptor agonist EC23 (Tocris, Bristol, UK) to support cultured submerged cells were until confluence; then, the cells were air-exposed by removing the apical medium and were differentiated by for 14 days in the same medium, with a higher concentration of EC23 (50 nM). Trans-epithelial electrical resistance [TEER >500 Ω∙cm2], cilia beating, and mucus secretion were assessed as markers of differentiation (Di Vincenzo et al., 2022). When the S-PBEC have reached the confluence and the ALI-PBEC completed the differentiation, cells were pre-treated with the NAC alone (1 mM) and with the antioxidant MIX (Curcumin 5μM, Vitamin B2 10 μM, NAC 1mM, Carnitin 1 mM) for 2 h, then was added CSE 20%. We used these concentrations based on previous publication (Vanella et al., 2017). Cells, basal medium and apical wash were collected after 24 h.

### 2.2 Preparation of cigarette smoke extract (CSE)

Cigarette smoke extract (CSE) was obtained using a peristaltic pump Watson-Marlow 323 E/D (Rotterdam, Netherlands) using two Kentucky 2R4F research-reference cigarettes (The Tobacco Research Institute, University of Kentucky) without filter. Briefly, each cigarette was smoked for 5 min in 20 mL of PBS to generate a CSE-PBS solution. The CSE solution was then filtered using a 0.22 μm pore filter to remove bacteria and large particles. This solution was considered 100% concentration. S-PBECs were stimulated with a concentration of 20% of CSE for 24h, while in the ALI-PBEC the 20% of CSE was added in the apical side for 30 min and then removed.

### 2.3 Reagents

Curcumin, Vitamin B2, L-Carnitin and N-acetyl-L-cysteine (NAC) were supplied, in powder form, by a pharmaceutical company (Nuova Farmaceutica, Catania) and solubilized in distilled water or a mix of water and DMSO (50% w/w) to obtain the working solutions. The new formulation was indicated as antioxidant mix (MIX).

### 2.4 Measure of mitochondrial superoxide (MitoSOX)

To evaluate the production of mitochondrial superoxide was used the MitoSOX™ Red mitochondrial superoxide indicator (Molecular Probes Waltham, MA, USA). After 24 h of stimulation, the cells were harvested, washed with PBS and stained with 3 μM MitoSOX Red probe for 15 min at 37°C. Then, PBECs were washed twice in PBS and then analyzed by flow cytometry using CytoFLEX (BeckmanCoulter). Analysis was done on 10,000 acquired events for each sample. Cell debris and dead cells were excluded from the analysis. Data were analyzed using the CytExpert software. The results were expressed as MFI (Mean Fluorescence Intensity).

### 2.5 Real time PCR analysis

The total RNA was isolated from PBECs using TRIzol Reagent (Life Technologies) after 24 h of stimulation, following the manufacturer’s instruction. 1 μg of RNA was reverse-transcribed to cDNA, using iScript cDNA Synthesis kit (Biorad). Gene expression of IL-8 and IL-6 was evaluated by qRT-PCR conducted at QuantStudio™ 3 Real-Time PCR System (Thermo Fisher Scientific, Waltham, MA, United States) using specific FAM-labeled probe and primers (prevalidated TaqMan Gene expression assay for IL-8, Hs00174103_m1; for IL-6, Hs00985639_m1, Thermo Fisher Scientific). GAPDH (prevalidated TaqMan Gene expression assay for GAPDH, Hs03929097_g1) was used as endogenous control gene to normalize the expression of the genes. The relative quantitation of mRNA was carried out with the comparative Ct method (2^ΔΔCt) and was plotted as respective fold-change. Untreated cells (NT) were used as reference sample.

### 2.6 ELISA assay

The release of IL-8 and IL-6 was evaluated by enzyme-linked immunosorbent assays (ELISA) (DuoSet R&D Systems, Minneapolis, MN) on PBECs. The basal medium of both S-PBEC and ALI-PBEC was collected, centrifuged at 12,000 *g* for 5 min, and then transferred to clean microcentrifuge tubes to be stored at −20°C for the subsequent analysis. To obtain the apical supernatant of ALI-PBEC, 250 μL of PBS was dispensed to each well and incubated at 37°C for 30 min. The PBS was collected in microcentrifuge tubes and stored at −20°C until use.

### 2.7 Observational clinical study

Fifty subjects were recruited at “Ospedali Riuniti Villa Sofia–Cervello”. The study was approved by the reference “Ospedali Riuniti Villa Sofia–Cervello” Ethic Committee (n. 221 AOR 2022) in accordance with the Declaration of Helsinki. Informed written consent was obtained from each subject. The COPD patients (N = 40) who meet the inclusion criteria, listed in Table 1, were recruited for the initial transversal study. A control group (N = 10) of healthy people was also included. Exclusion criteria were considered exacerbated COPD patients requiring systemic corticosteroids and/or antibiotic therapy, positive skin tests for common aeroallergen extracts and history of asthma and/or allergic *rhinitis.* Regarding comorbidities in COPD patients, the most prevalent (75%) was arterial hypertension under treatment, followed by dyslipidemia (25%), chronic ischemic heart disease (12.4%) and Type 2 diabetes mellitus (6.2%). Among forty COPD patients, only sixteen chose to carry out the 6 months observational study No significant differences were found for cigarette pack/year, Forced Expiratory Volume in 1 s (FEV1) L/%, Forced Vital Capacity (FVC) L/%, Body Mass Index (BMI), within the COPD patient group (Table 2).

**TABLE 1:** Patients’ inclusion criteria.

Inclusion criteria
current and former smokers with > 10 pack-week
smoking history ≥ 30 years
Age > 45
established COPD diagnosis
number of COPD exacerbations > 2

**TABLE 2:** Patients’ demographics.

	Controls	COPD patients
*N*	10	40
*Age*	49 ± 2	67 ± 7
*Gender (M/F)*	(7/3)	(27/13)
*GOLD*	-	1-4
*BMI*	25 ± 2	25,9 ± 3

### 2.8 Study design

Open prospective observational study, to assess the effects of a new nutraceutical formulation based on curcumin, carnitine, acetyl-N-cysteine-L, vitamin B2, piperine added to traditional therapy for patients with COPD. Sixteen patients with COPD agreed to take the new nutraceutical formulation every day for 6 months in addition to background therapy (LAMA + LABA + ICS). The subjects recruited for the study were evaluated at visit 1 (V1) (before administering the new nutraceutical formulation) and at visit 2 (V2) (after 6 month) for clinical and functional assessment (COPD Assessment Test (CAT) questionnaire, spirometry and walking test) and for nasal wash evaluations (IL-8).

### 2.9 Nasal wash sampling

Nasal washes were obtained from healthy controls and from COPD patients. Samples were obtained by irrigation of each naris with 9 mL of saline through a rinowash (Air Liquide Medical Systems). The fluid was collected in the external tank of the instrument, then transferred in a conical polypropylene tube and stored at 4°C until the processing in laboratory. The volumes of nasal washes collected were measured and Dithiothreitol (DTT) (Sputolysin, Calbiochem Corp., San Diego, CA, United States), freshly prepared in a 10% dilution with distilled water, was added to each sample in the equivalent volume to one-10th. Samples were vortexed to homogenization and centrifuged at 500 *g* for 10 min at 4°C. After centrifugation, the supernatant was collected and stored at −80°C for the subsequent analysis.

### 2.10 Evaluation of clinical and functional parameters

The recruited COPD patients treated with new nutraceutical formulation were evaluated at V1 and V2 for symptoms and functional parameters. The functional parameters, Forced Expiratory Volume in 1 s (FEV1) and Forced Vital Capacity (FVC), were evaluated by (SensorMedics Vmax) spirometry. Spirometry was performed in accordance with the American Thoracic Society/European Respiratory Society (ATS/ERS) guidelines (Graham et al., 2019). The functional parameters of this subjects at V1 were: FEV1 L: 1.33 ± 1.05; FEV1%: 63 ± 31, FVC L: 2.32 ± 1.33; FVC %: 85 ± 24.5; tiff: 0.6 ± 0.18. After 6 months of treatment, at V2, the functional parameters did not undergo any significant changes (FEV1 L: 1.27 ± 1.17; FEV1%: 63 ± 31, FVC L: 2.35 ± 1.6; FVC %: 82 ± 31.5; tiff: 0.6 ± 0.14). Furthermore, patient symptoms were collected using the COPD Assessment Test (CAT) questionnaire.

### 2.11 Six-minute walking test (6MWT)

The 6MWT was performed during the first visit and at the end of the 6 months observation period, according to the American Thoracic Society (ATS) guidelines (Enright, 2003). Briefly, patients were asked to walk for 6 min back and forth between two cones for a total of 30 m. Saturation was detected during the test. Data of the distances covered were recorded in percentage of meters walked by the patients.

### 2.12 Statistical analysis

Data from PBEC culture were expressed as mean ± SD and analyzed by using ANOVA test (Fisher’s). At *p*-value < 0.05, differences were considered to be statistically significant. Data from patients were analyzed by using Mann–Whitney test or Wilcoxon test. At *p*-value < 0.05, differences were considered to be statistically significant.

## 3 Results

### 3.1 Release of mitochondrial superoxide

**FIGURE 1:**
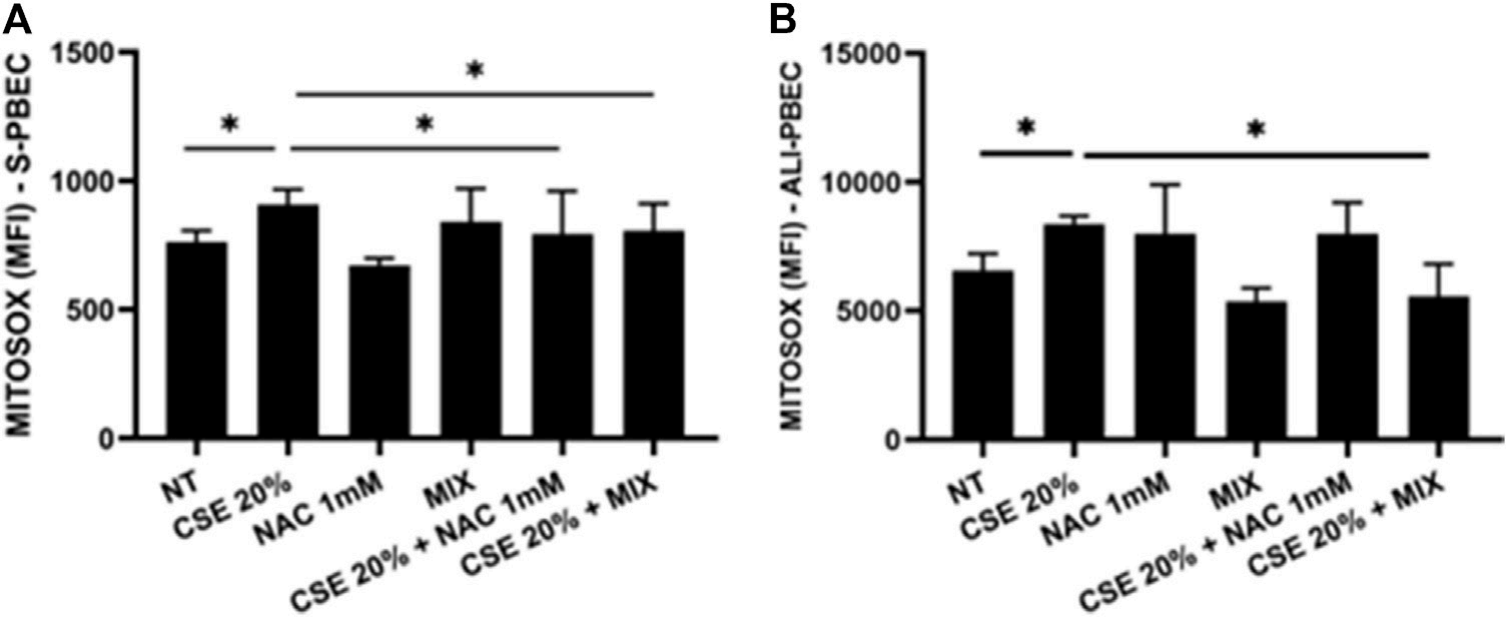
Production of mitochondrial superoxide in S-PBEC and in ALI-PBEC. S-PBEC **(A)** and ALI-PBEC **(B)** were pre-treated with NAC or MIX for 2 h and then stimulated with CSE 20%. After 24 h, mitochondrial superoxide production was evaluated by flow cytometry. Data are showed as Mean Fluorescence Intensity ±SD (S-PBEC, N = 6; ALI-PBEC, N = 6), *p < 0.05. ANOVA (Fisher’s) test.

To understand whether the new formulation of N-acetylcisteine (NAC), carnitine, curcumin and B2 vitamin could exert beneficial effects against cigarette smoke damage, we assessed the production of mitochondrial superoxide, the main ROS produced by the damage to the respiratory chain, in S-PBEC and ALI-PBEC. The results showed that CSE significantly increased the production of mitochondrial superoxide in both cellular models (Figures 1A, B). Pre-treatment with the new formulation, indicated as MIX, significantly counteracted the effect of CSE on oxidative stress (Figures 1A, B) in both types of PBEC culture. Pre-treatment with NAC alone significantly counteracted the effect of CSE on oxidative stress (Figures 1A, B) in S-PBEC.

### 3.2 IL-8 and IL-6 gene expression and release

**FIGURE 2:**
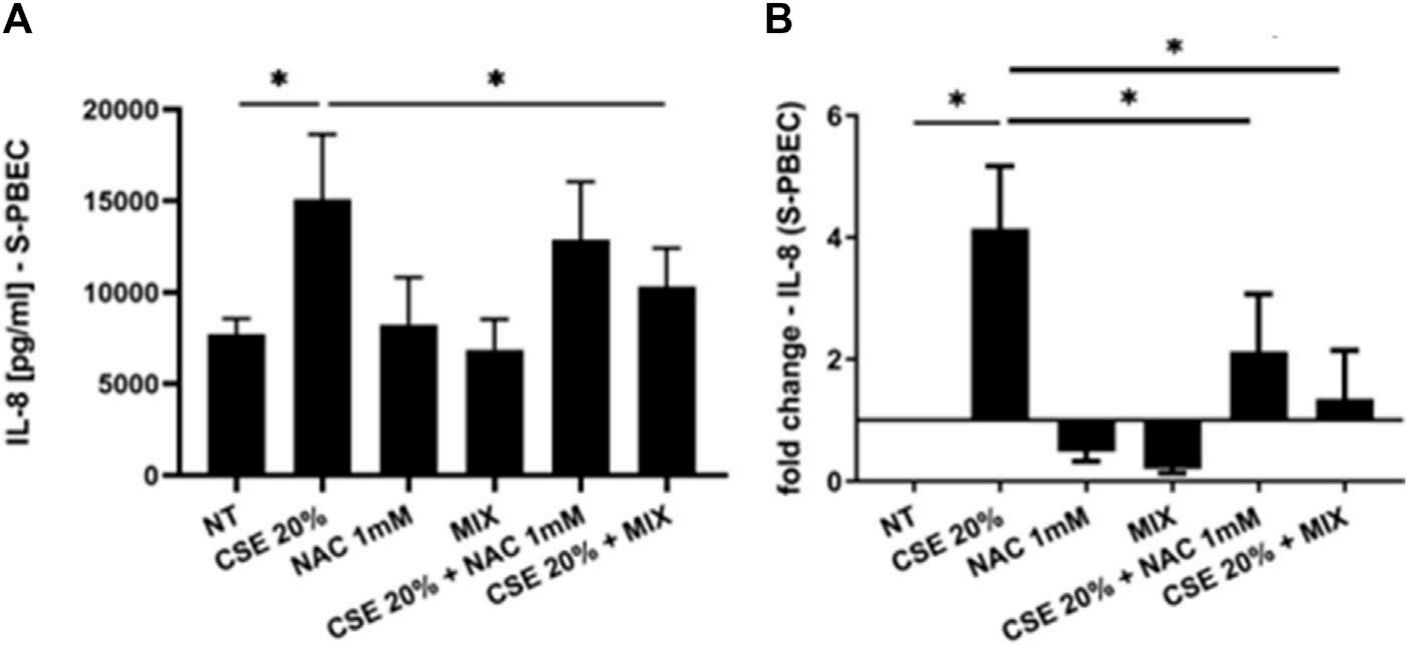
IL-8 release and gene expression in S-PBEC. PBECs were pre-treated with NAC or MIX for 2 h and then stimulated with CSE 20%. After 24 h, the medium was collected and total RNA was extracted to evaluate IL-8 protein release and gene expression, respectively. **(A)** IL-8 release measured by ELISA. Results are reported as pg/mL (N = 9). *p < 0.05 ANOVA (Fisher’s) test. **(B)** IL-8 gene expression measured by RT-PCR. Results are reported as mean fold change compared to control ±SD (N = 8). *p < 0.05 ANOVA (Fisher’s) test.

**FIGURE 3:**
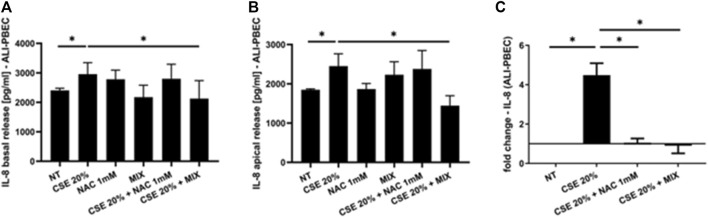
IL-8 basal and apical release and gene expression in ALI-PBEC. PBECs differentiated using ALI-culture were pre-treated with NAC or MIX for 2 h and then stimulated with CSE 20%. After 24 h, basal medium and apical wash were collected and total RNA was extracted to evaluate IL-8 protein release and gene expression. **(A)** IL-8 basal release and **(B)** apical release measured by ELISA. Results are reported as pg/mL (N = 9). *p < 0.05 ANOVA (Fisher’s) test. **(C)** IL-8 gene ex-pression measured by RT-PCR. Results are reported as mean fold change compared to control ±SD (N = 8). *p < 0.05 ANOVA (Fisher’s) test.

**FIGURE 4:**
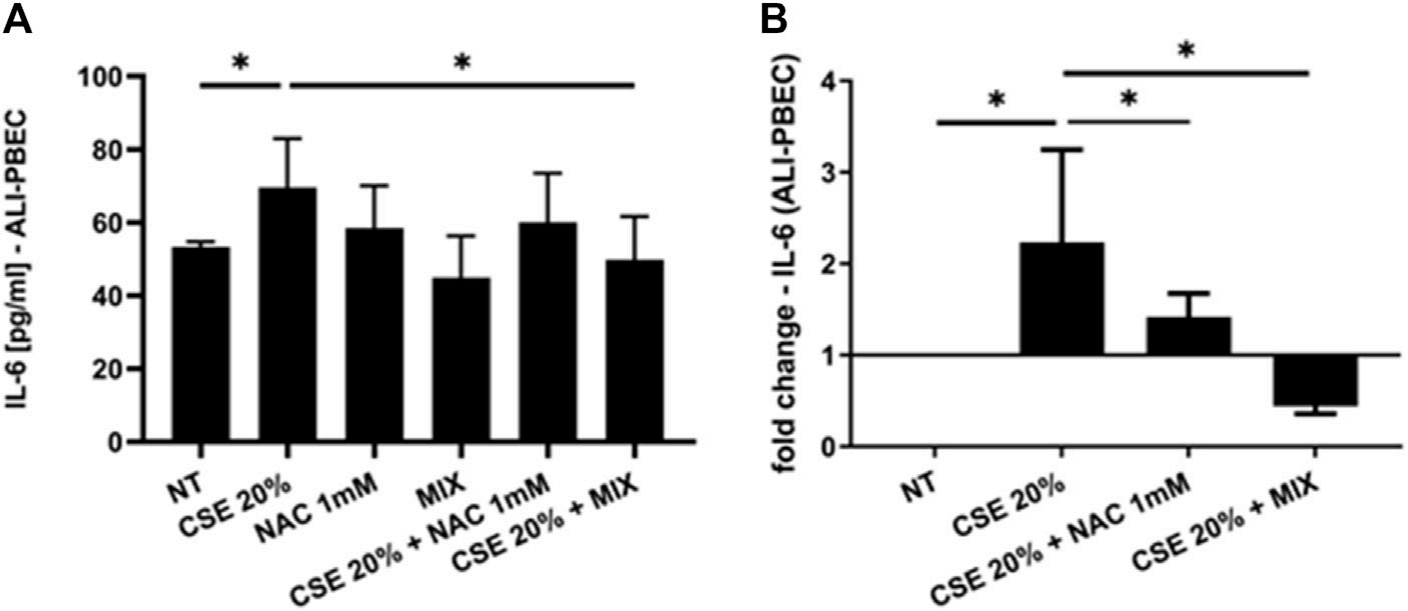
IL-6 basal release and gene expression in ALI-PBEC. PBECs differentiated using ALI-culture were pre-treated with NAC or MIX for 2 h and then stimulated with CSE 20%. After 24 h, basal medium was collected and total RNA was extracted to evaluate IL-6 protein release and gene expression. **(A)** IL-6 protein release measured by ELISA. Results are reported as pg/mL (N = 9). *p < 0.05 ANOVA (Fisher’s) test. **(B)** IL-6 gene expression measured by RT-PCR. Results are reported as mean fold change compared to control ±SD (N = 8). *p < 0.05 ANOVA (Fisher’s) test.

Since increased oxidative stress leads to amplified inflammatory responses (Bodas et al., 2017), we evaluated IL-8 gene expression and release in PBECs in both S-PBEC (Figures 2A, B) and in ALI-PBEC (Figures 3A, B). In S-PBEC, CSE increased the IL-8 gene expression and release and the MIX significantly counteracted CSE mediated effects on both IL-8 gene expression and release (Figures 2A, B) while NAC exerted counteracting activity only on IL-8 gene expression (Figure 2B). In ALI-PBEC, CSE increased the IL-8 gene expression and basal and apical IL-8 release and the MIX significantly counteracted CSE effects on both IL-8 gene expression and apical and basal release (Figures 3A–C) while NAC exerted counteracting activity only on IL-8 gene expression (Figure 3C). IL-6 gene expression and release was also evaluated in ALI-PBEC (Figures 4A, B). The results showed that CSE in-creased IL-6 gene expression and basal release (Figures 4A, B). No apical IL-6 release was detected. MIX significantly reduced IL-6 gene expression and basal release (Figures 4A, B). NAC alone reduced only the IL-6 gene expression. No significant results were obtained for the gene expression and release of IL-6 in S-PBEC (data not shown).

### 3.3 IL-8 in nasal wash from control subjects and COPD patients pre- and post-therapy with the nutraceutical drug

**FIGURE 5:**
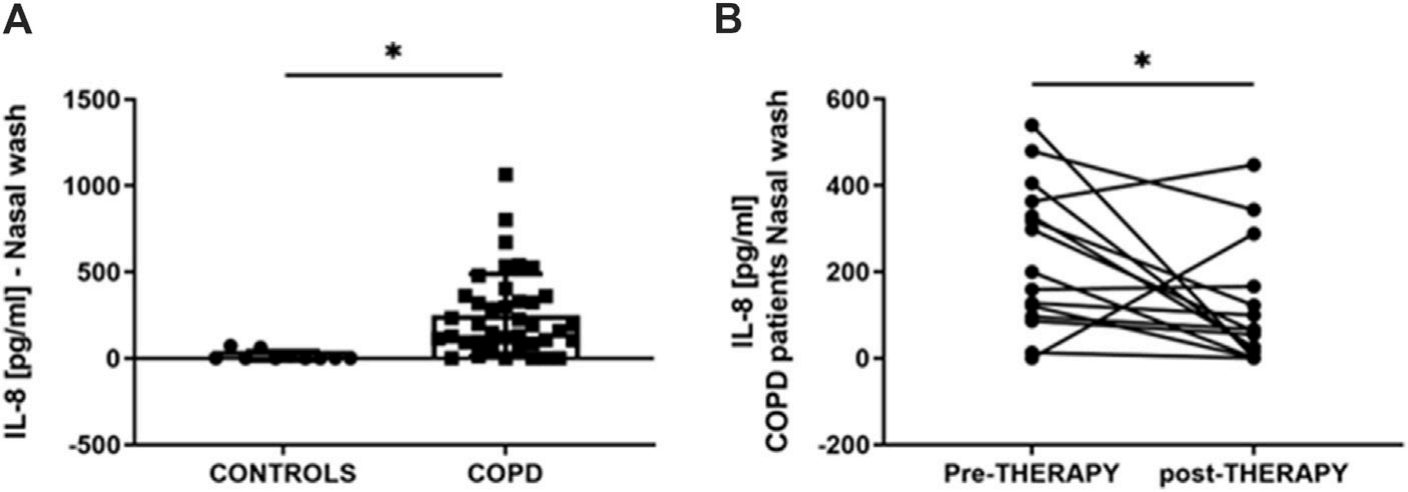
IL-8 in nasal wash from control subjects and COPD patients pre- and post-therapy with antioxidant drug. IL-8 protein measured by ELISA in nasal wash from Controls and COPD patients. Results are reported as pg/mL. *p < 0.05 Mann-Whitney test **(A)**. Comparison of IL-8 levels pre-and post-nutraceutical treatment intra-patient. Results are reported as pg/mL. *p-value < 0.05 Wilcoxon test **(B)**.

**FIGURE 6:**
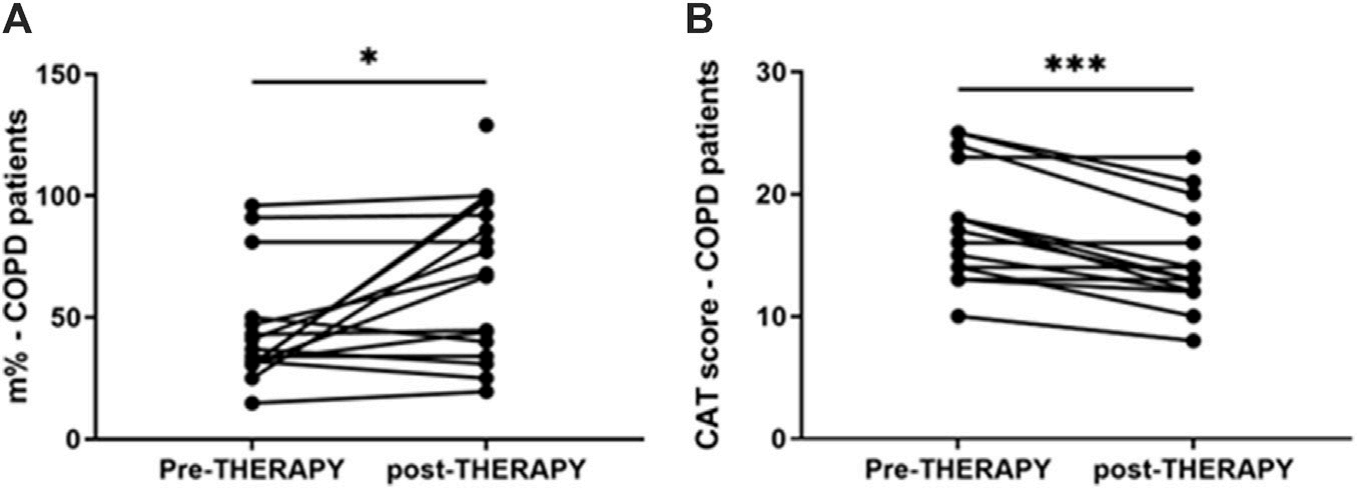
Walking test and CAT in COPD patients pre- and post-therapy with antioxidant drug. Walking test on COPD patients was performed at V1 and V2, after 6 months. Results are reported as distance in percentage of meters that patients quickly walked on a flat surface in 6 min *p-value < 0.05 Wilcoxon test **(A)**. CAT score in COPD patients at V1 and V2, after 6 months. CAT values reported for individual patients. ***p-value = 0.001 Wilcoxon test **(B)**.

IL-8 levels were assessed in nasal wash from controls (n = 10) and COPD patients (n = 40). As shown in Figure 5A, IL-8 nasal wash was significantly higher in COPD than in Controls. To establish whether the new nutraceutical formulation was effective *in vivo* in modulating IL-8 nasal wash levels, an observational prospective study on COPD patients (n = 16), treated or not with the new nutraceutical formulation for 6 months, was conducted. Obtained data demonstrated that 6 months treatment with the new formulation significantly reduced nasal wash IL-8 levels (Figure 5B). Moreover, in COPD treated patients a significant improvement in walking test (Figure 6A) as well as in CAT score data (Figure 6B) was observed. The results of the study power calculations on these three outcomes to evaluate the effectiveness of the tested MIX are reported on Table 3.

**TABLE 3:** Power Study Results: Effect Size and Statistical Power for the outcomes IL-8, CAT Scores e walking test.

Outcome	Effect size	Power
IL-8 in nasal wash	0.231	0.139 (13.9%)
CAT score	−1.303	0.997 (99.7%)
Walking test	0.666	0.702 (70.2%)

## 4 Discussion

Cigarette smoking is the main cause of increased oxidative stress in the airways of COPD patients. The aim of our work was to assess whether a new formulation containing a MIX of substances, N-acetylcisteine (NAC), carnitine, curcumin and B2 vitamin, with recognized antioxidant properties would be more effective in counteracting the effects of cigarette smoke on epithelial cells. In details, we used a complex *in vitro* model of human primary bronchial epithelial cells differentiated to form a pseudostratified epithelium like the real airway epithelium. Our experimental models are based on the use of both a 2D model of submerged/undifferentiated primary bronchial epithelial cells (S-PBEC) and a 3D model differentiated cells (ALI-PBEC). Treatment exposures in S-PBECs are significant but experimentally simpler. The use of ALI-PBECs could physiologically represent a more realistic model and thus potentially more meaningful from a biological point of view. Furthermore, this model represents a fundamental resource that likely reflects inherent biological heterogeneity in terms of function (Lenz et al., 2013). Indeed, ALI-PBEC models represent a fully differentiated epithelium with more than 1 cell type (ciliated cells, goblet cells, and basal cells, etc.) that can also be combined with immunocompetent cells (dendritic cells, macrophages, neutrophils) (Upadhyay and Palmberg, 2018). However, in our study the use of ALI-PBEC as experimental model provides further insight into the pathogenesis of COPD, since they more closely approximate the physiological cell state *in vivo* compared to stable cell lines. Exposure to cigarette smoke causes the structural and functional alteration of the mitochondria at the level of the epithelium, with a consequent increase in oxidative stress (Hoffmann et al., 2013). The evaluation of mitochondrial ROS, compared with untreated cells, was an indication of the mitochondria functionality and homeostasis. Our results showed an increased production of mitochondrial ROS induced by CSE compared to untreated cells. In ALI-PBECs, pre-treatment with the MIX of antioxidants but not with NAC significantly reduced the amount of mitochondrial superoxide induced by CSE. NAC alone pretreatment was able to counteract the effects of CSE in mitochondrial superoxide production in S-PBEC confirming recent studies (Son et al., 2020; Hur et al., 2022) that have highlighted antioxidant properties of NAC, thanks to its ability to regenerate glutathione in similar submerged models. However, the use of additional substances such as curcumin, carnitine and vitamin B2, already widely used as a supplement (Patel et al., 2020; Suwannasom et al., 2020), can have a synergistic effect and greater therapeutic efficacy, restoring the mitochondrial homeostasis in a model closer to real airway mucosa. Notably, a previous report tested the similar antioxidant MIX in the cell line 16HBE (Vanella et al., 2017). Furthermore, our study evaluated the potential anti-inflammatory effects of the mixture in relation to both the gene expression and release of inflammatory cytokines and chemokines (IL-6 and IL-8, respectively) by the ALI-PBEC, following the exposure to CSE. The use of ALI-PBECs, compared to the continuous immortalized cell lines, allowed the analysis of the two different cellular compartments, basal and apical. Of particular importance are the data relating to the expression and release of IL-8, which had not been evaluated previously. It is worth noting that cigarette smoke causes abnormal airway inflammation, triggering the release of chemokines and promoting the infiltration of neutrophils and other inflammatory cells into the airways (Ma et al., 2023). Several studies reported an increase of IL-8 levels induced by cigarette smoke (Pace et al., 2008; Moon et al., 2013). IL-8 is a key mediator of inflammation response, with its chemoattractant capability, and it is released by several cell types, including epithelial cells (Brennan and Zheng, 2007). In particular, it is well known that IL-8 is a potent neutrophil chemoattractant chemokine. Neutrophil accumulation in the lung is a prominent feature of COPD. The accumulation occurs in the lung tissue of COPD patients and it is associated with alveolar damage and lung dysfunction (Williams and Jose, 2000). Some studies have found a correlation between IL-8 levels, neutrophil counts, markers of neutrophil activation, such as myeloperoxidase activity, and the degree of lung dysfunction (Jasper et al., 2019). Our results showed that ALI-PBEC, when exposed to CSE, released more IL-8 in both apical and basal side. These effects were well-counteracted by the pre-treatment with the antioxidant MIX, which led to an important reduction of IL-8 in both compartments. Also in S-PBEC, CSE increased IL-8 release, and MIX significantly counteracted the CSE-mediated effects of IL-8. This confirms that cigarette smoking could play a role in the airway and systemic neutrophilia characteristic of COPD in fact the release of IL-8 is elevated both towards the lumen of the respiratory tract (apical side) and towards the underlying tissue (basal side). Accordingly, here, the concentration of IL-8 in nasal lavage is significantly higher in COPD patients than in control subjects, highlighting the increased airways inflammatory state of COPD patients. Since COPD patients often experience infective based exacerbation, in ALI-PBEC the effects of both CSE and MIX on IL-6 gene expression and protein release were also assessed. IL-6 is a cytokine produced in the acute phase of inflammation in response to infections and other inflammatory stimuli, including CSE (Dawson et al., 2021; Krick et al., 2021). Several studies reported elevated levels of IL-6 in patients with respiratory diseases, such as asthma and COPD, in which the inflammatory status and the lung epithelial damage are well known (Rincon and Irvin, 2012; Bradford et al., 2017). Furthermore, an interesting association between high levels of IL-6 and increased mortality or poor prognosis of patients was found, since IL-6 plays a key role in the pathogenesis of these diseases and heavily alters lung homeostasis (Agustí et al., 2012; Ilmarinen et al., 2016). Our results in ALI-PBEC showed an increase of IL-6 basal release, when stimulated with CSE and pre-treatment with the antioxidant MIX led to a significant reduction of the gene expression levels and the release of the inflammatory cytokine, indicating a potential restoring of airway homeostasis. The emergence of Precision Medicine revolutionized the approach of translational research. Reason being, with our study we have sought to support and contribute to the development of patient-centered translational medicine by translating what has been basic research into what could be called clinical pre-experimentation. The clinical benefits and anti-inflammatory effects of a new nutraceutical formulation, containing curcumin, carnitine, acetyl-N-cysteine-L, vitamin B2, and piperine, were also evaluated in patients with COPD in an observational study before and after 6 months of treatment with this nutraceutical drug, similar to the MIX used in the *in vitro* models. An interesting result was obtained from the comparison between control subjects and COPD patients in nasal lavage. Indeed, it was shown that the concentration of IL-8 is significantly higher in patients, highlighting the normal inflammatory state that characterizes the latter. The concentration of IL-8 in the nasal washes of COPD patients after 6 months of treatment with the new nutraceutical formulation is reduced compared to before the therapy. The progressive obstruction of the airways typical of COPD patients leads to dyspnea, i.e., shortness of breath during exertion and, therefore, can cause patients limitations of physical exercise (Kerti et al., 2018). In light of this, during our observational study the evaluation of patients’ physical effort tolerance through the walking test were performed. The results showed a significant increase in the percentage of meters walked by patients during the test, demonstrating an effective improvement of the patient’s physical abilities. These results are also in agreement with those obtained from the COPD Assessment Test or CAT score. This test easily assesses the impact of COPD symptoms such as cough, dyspnea, sputum, and chest tightness on patients’ health status. The CAT score ranges from 0 to 40 and the higher the score, the more severe the impact of COPD on the patient’s life. Our intra-patient results evidenced a significant reduction of the CAT score after 6 months of the treatment with the nutraceutical formulation, reflecting an effective general improvement in the patient’s health conditions (Gil et al., 2021). The power study results showed that the evaluation of the IL-8 in nasal washes of patients, pre- and post-therapy, has a low power level (13.9%). This means that to achieve an acceptable power level, the sample size or effect size needs to be increased. However, for two of the outcomes of interest (i.e., the CAT score and walking test improvements) we obtained, respectively, a high (99.7%) and moderate (70,2%) power level, indicating that the study is very likely to detect a true effect. Thus, for the first time, this study demonstrated and further confirmed the beneficial effect and the protective role of the antioxidant and anti-inflammatory MIX in a complex model and could be employed as supportive therapy that could help prevent any relapses in COPD patients.

Our *in vitro* study demonstrated the efficacy of a novel antioxidant formulation against the effects of CSE in both S-PBECs and ALI-PBECs. Specifically, pretreatment with the antioxidant MIX counteracts the effects of CSE on oxidation and markers of inflammation, restoring cellular homeostasis. Moreover, inflammatory cytokines such as Thymic stromal lymphopoietin (TSLP), IL-33 and Transforming growth factor beta (TGF-β) were also investigated in our *in vitro* study, but we did not obtain significant results (data not shown). However, further inflammatory cytokines (i.e., Tumor necrosis factor alpha (TNF-α) and antioxidant mechanisms could be explored in future studies. It would be also interesting to study the effect of the antioxidant MIX on the activation of damaged respiratory epithelium on cells of the innate and adaptive immune system. Interestingly, the observational study showed interesting preliminary data on the use of MIX antioxidant. The case is very interesting in the context of translational medicine that is increasingly focused on patient wellbeing. This study could provide the interesting basis for larger clinical studies and to further validate the antioxidant and anti-inflammatory effect of this nutraceutical on symptoms and on the prevention of periodic exacerbation in COPD patients. However, this study presents limitations. Since it is designed as a pilot study and we observed a small number of patients, there is the lack of a control group (placebo) to which compare the effectiveness of the MIX regarding the outcomes of our interest, such as changes in inflammatory cytokines. Furthermore, only 16 patients completed the study. The reasons for patient dropout were varied, including difficulty in dissolving the MIX power in water, poor palatability of the nutraceutical, and, in some cases, side effects such as abdominal pain and diarrhea. However, these aspects will certainly be addressed and improved in future large-scale clinical studies.

## Data Availability

The original contributions presented in the study are included in the article/Supplementary Material, further inquiries can be directed to the corresponding author.
