# Supplementary material for: In vitro evidence of antioxidant and anti-inflammatory effects of a new nutraceutical formulation explains benefits in a clinical setting of COPD patients
Source: Front Pharmacol. 2024 Aug 20;15:1439835. doi: 10.3389/fphar.2024.1439835 (PMC11368797; doi:10.3389/fphar.2024.1439835)
Supplement: Supplementary file 1 [file DataSheet1.docx]

Supplementary Material

**Cell viability assay**

Cell viability was evaluated by CellTiter 96 Aqueous One Solution Cell Proliferation Assay, (PROMEGA, Madison WI USA) according to the manufacturer’s instructions. One Solution reagent contains MTS [3-(4,5-dimethylthiazol-2-yl)-5-(3-carboxymethox-yphenyl)-2-(4-sulfophenyl)2H-tetrazolium].

Submerged Primary Bronchial Epithelial Cells (S-PBECs) were seeded in a 96-well plate and treated with Curcumin 5μM, Vitamin B2 200 μM and 10 μM, NAC 1 mM and Carnitin 1mM, the antioxidant MIX (Curcumin 5μM, Vitamin B2 10 μM, NAC 1mM, Carnitin 1mM) and DMSO. After 24 hours of treatment, 20 μL of One Solution reagent was added to each well and incubated for 20 min at 37 °C with 5% CO_2_. The absorbance was read at 490 nm using a Microplate reader Wallac Victor 2 1420 Multilabel Counter (Perkin Elmer, Milan, Italy). Results are expressed as percentage of viability compared with untreated cells (100% viability).

# Supplementary Figures


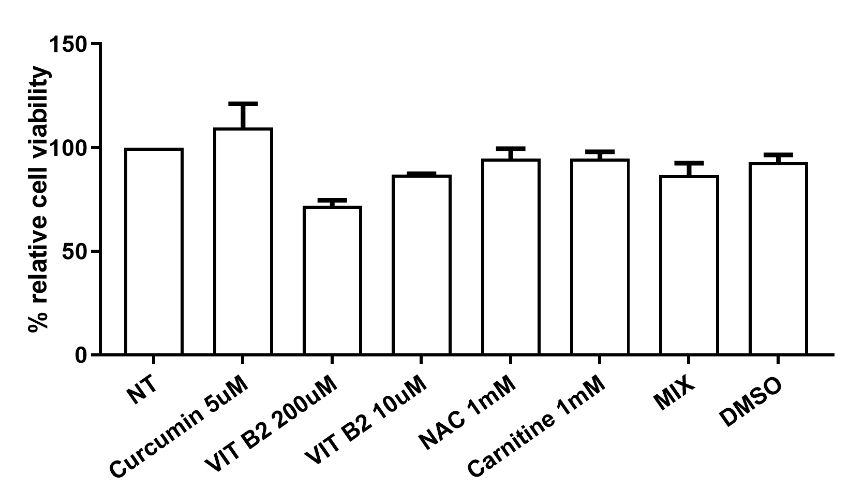


**Supplementary Figure 1.** Effects of on cell viability/metabolism in S-PBECs treated with Curcumin 5μM, Vitamin B2 200 μM and 10 μM, NAC 1mM and Carnitin 1mM and the antioxidant MIX. Results are expressed as percentage relative to no treated (NT) sample. Data represent mean ± SD (n=3).
